# Ontological analysis of SNOMED CT

**DOI:** 10.1186/1472-6947-8-S1-S8

**Published:** 2008-10-27

**Authors:** Gergely Héja, György Surján, Péter Varga

**Affiliations:** 1 National Institute for Strategic Health Research, Arany János utca 6-8, 1051 Budapest, Hungary; 2 Eötvös Loránd University, Egyetem tér 1-3, 1053 Budapest, Hungary

## Abstract

**Background:**

SNOMED CT is the most comprehensive medical terminology. However, its use for intelligent services based on formal reasoning is questionable.

**Methods:**

The analysis of the structure of SNOMED CT is based on the formal top-level ontology DOLCE.

**Results:**

The analysis revealed several ontological and knowledge-engineering errors, the most important are errors in the hierarchy (mostly from an ontological point of view, but also regarding medical aspects) and the mixing of subsumption relations with other types (mostly 'part of').

**Conclusion:**

The found errors impede formal reasoning. The paper presents a possible way to correct these problems.

## Background

The National Institute for Strategic Health Research (from here on referred to as ESKI) is evaluating SNOMED CT [1] to be used in the Hungarian health care sector. It could be used for the following purposes:

• A common reference terminology for classification systems (ICD10 [2], Hungarian adaptation of ICPM [3])

• Providing a common resource for enabling the interoperability of healthcare information systems in Hungary

• Enabling interoperability with the healthcare systems of other EU member states. This goal – at least – requires the extension of the previous terminology to a multilingual conceptual system. In this paper we do not focus on this task.

SNOMED seems to be the first choice candidate because it is the most comprehensive clinical terminology system. This paper examines whether SNOMED contains ontological errors which would prevent the achievement of these goals.

The first purpose does not require an exhaustive list of all possible medical concepts, rather "a set of building blocks and constraints from which concepts can be composed" [4]. Consequently, we do not want to find the exactly matching concept to a given category of the classification system (which is practically not possible), but find those concepts from which the category can be composed. This approach is similar to that of SNOMED International [5]. Since these classification systems represent only a small part of the medical knowledge (e.g. diseases, procedures) the representation of their categories requires only a subset of the concepts of SNOMED CT. The formal definition of the categories of these classification systems enable formal consistency checking (aiding maintenance), (semi)automatic interconnection of the classification systems (e.g. creating rules between diseases and procedures, or mapping the Hungarian version of ICPM to the procedure classification of ICD9-CM) and supporting statistical analysis (by determining the appropriate categories to a query, e.g. "injuries of hand"). These services require that the resulting conceptual system is suitable for automatic reasoning, which can only be achieved if the core ontology is suitable for it.

The support of semantic interoperability [6] requires a detailed common terminology (or ontology). The reason for this is that the current healthcare information systems and communication standards frequently use pre-coordinated lists, which may cover any domain of medical knowledge. These concepts used by the healthcare information systems have to be mapped to those of a common terminology, and vice versa. This mapping requires a common, consistent, comprehensive and decidable ontology. Automatic reasoning can efficiently support the ensuring of consistency. However, comprehensiveness and computability (i.e. decidable within a reasonable time) are two contradictory requirements. Consequently, a practical compromise has to be found. A consistent ontology should insist on the separation of subsumption (is a) from other relations (e.g. part of, acts on) and rely on formal definitions in addition to simple taxonomic subclass hierarchies.

Machine reasoning is a task of at least exponential time. Therefore, reasoning about a system containing approximately 350 thousand concepts is a problem not yet practically solved. Conceptual systems used for automated reasoning require a different structure with less emphasis on coverage (the multitude of non-defined leaf categories) and greater emphasis on rich and well-organized high-level categories. Whilst SNOMED seems to be a terminology of high coverage, which this paper does not address, it is to be investigated whether it enables automatic reasoning.

## Methods

We used the January 2006 release of SNOMED using the Clue Browser 5.5 in our analysis. We reviewed several concepts selected arbitrarily, both high level abstract concepts (such as clinical finding) and low-level concrete concepts (e.g. *pneumonia*, *smoker *and *acute*) from the domains of ESKI (diseases, risk factors). The selection was biased: we selected concepts which are prone to be represented incorrectly (e.g. roles) and show the typical error of representing relations as subsumption. The following analysis steps have been performed on these concepts:

• Is the concept really subsumed by its explicit parent concept(s) and does it subsume its children? This analysis is based on two examinations: can we classify both concepts into the same category of the DOLCE upper level ontology, and is it true that their relation is subsumption and not an other relation (e.g. part of, plays) [7,8]. We assumed that the intended meaning of the categories is reflected by the preferred term.

• Does the meaning derived from the preferred term concur with the meaning derived from the synonyms and formal definition?

DOLCE (Descriptive Ontology for Linguistic and Cognitive Engineering), a descriptive upper-level ontology, is especially designed for automatic reasoning and interoperability [9]. It has taken several notions from OntoClean [10], a methodology created for ontology analysis based on the metaproperties of classes. In contrast to certain other efforts aiming at the same tasks, such as SUMO [11], it is based on sound theoretical foundations, which made it well suited for our purposes.

DOLCE is conceived as a descriptive ontology (i.e. aiming at describing in a coherent way our use of concepts, not revising them) based on solid philosophical grounds. It incorporates, among others, a distinction between entities which extend through time (endurants) and which happen in time (perdurants). In order to achieve coherent descriptions, DOLCE often distinguishes between several entities co-locating in the same space and time (e.g. a statue and the clay that constitutes it).

DOLCE is augmented by ontology modules which together form the so called DOLCE-Plus. One of these modules, the Descriptions and Situations (ExtendedDnS), is of particular importance for us since it contains the DOLCE's abstraction of socially constructed entities (like finding etc.) There exists a transcription of DOLCE-Plus to the description logics formalism called DOLCE-Light-Plus.

## Results

### Errors identified based on the analysis criteria

After we have performed the analysis described in the previous section, the following types of ontological errors can be abstracted. We give examples for each type, together with the proposed revisions (SNOMED CT concept labels are in *italic*, while DOLCE category names in **bold**):

1. Hierarchy violating the rules of sound ontology engineering (inconsistent classification to DOLCE). For example *smoker *(intuitively it should be a kind of **agent**) is subsumed by *tobacco smoking behaviour – finding *(a **role**). DOLCE clearly distinguishes between a **role **(a socially constructed theoretical entity) and the **agent **(a physical entity), which plays that role. The subsumed concepts should have the same classification to DOLCE as the parent concept.

2. Mixing the subsumption relation with other relations (e.g. part of). For example *haemoglobin *subsumes *haemin*, while in fact haemin is a constituent of haemoglobin (both these concepts can be classified as **non-agentive physical objects**). Likewise *exacerbation of asthma attack *is subsumed by *asthma*, although it should be a temporal part of it (these concepts can be classified as **perdurant**). The relations which are not subsumptions should be removed from the hierarchy.

3. Hierarchy violating medical thinking and biomedical knowledge. *Disease*, *observation *and *finding *are subsumed by *clinical finding*, which is erroneous even from the medical point of view. Disease, finding, observation, and complaint are mutually disjoint medical concepts, consequently none of them should subsume one of the others. The DOLCE-ExtendedDnS sub-ontology helps us to distinguish betweens **situations**, their **descriptions **and the **concepts **used for describing them. We found that the categories describing disease courses also lack solid ground, consider *acute on chronic*, which is both subsumed by *acute *and *chronic*. Similarly, *severe asthma *is a kind of *asthma finding *in stead of *asthma*. The hierarchy should conform to medical knowledge but also to rigorous formal criteria [7].

4. Contracting disjoint entities into one concept. For example, *smoker *and *smoker (finding) *are synonyms. The first should be classified as an **agent, **while the latter seems to be a statement that the patient is a smoker which can be classified as a **description **(of a situation). Likewise, *additional pathologic finding in tumor specimen (observable entity) *has a synonym *additional pathologic finding* (however, it is possible that this problem is only caused by improper naming). *Function *is classified as an *observable entity*. However, under ontological scrutiny, function is an ability of an object to play a certain role in a certain kind of activity. *Function *in SNOMED subsumes both functions (in ontological sense, e.g. gene function or adaptation) and *measures *(**quality**) that evaluate the realisation of a function (e.g. *respiratory rhythm *or *excretory rate*). *Inflammation (morphological abnormality) *(**non-agentive-physical-object**) has a synonym *inflammatory reaction *(**perdurant**).

### Additional errors identified

Apart from these errors found by the described methodology, we have involuntarily found other problems with the system:

• Categories taken from classification systems. For example, *pneumonia in other diseases classified elsewhere *(marked as "ConceptStatus Limited") is clearly taken from a classification system, since no physician would write down such an expression. This example illustrates the dangers of taking over concepts from other conceptual systems: the context of a concept can get lost. In the original classification system it can be known what is meant by "other diseases classified elsewhere", but this knowledge is lost in SNOMED. In this form it clearly mixes a meta-statement about the representation with the content of the representation.

• Relations (called roles in DL, such as "part of") are also represented as concepts. This approach prohibits the direct conversion to any formalism based on first order logic, and, a fortiori, to any description logic formalism, such as OWL DL.

• Underspecification. The other cause which prohibits the automatic translation of the obtained distribution to a DL language is that the roles are not quantified. In each case it has to be decided whether to use existential or universal quantification.

• Common use of multiple inheritance, with frequent subsumption errors (mostly error type 1). For example, *alcoholic beverage *(through its parent *ingestible alcohol*) is subsumed by *central depressant*, *ethyl alcohol *and *psychoactive substance of abuse – non-pharmaceutical*. From a philosophical point of view none of these subsumptions is true. Alcoholic drinks contain ethyl alcohol which plays a role of depressant and substance of abuse (with respect to human beings). It is likely that most polyhierarchies in SNOMED follow this pattern. Consequently, they should be revised, and if necessary eliminated.

## Discussion

The ontological errors identified by our analysis may be caused by the fact that SNOMED CT has been developed by giving high priority to the needs of clinicians: i.e. to find easily the concepts they search for. In this context the ontological errors such as ethyl alcohol subsuming alcoholic beverage is a minor problem. However, the frequent occurrences of these errors challenge the rationality of automatic reasoning. On the other hand, it would have been possible to create a conceptual system on sound philosophical principles (to assist formal reasoning and consistency checking) and later transform it to a more end-user-oriented format.

As a consequence of the listed error types, the intended meaning of the categories is not always clear. Sometimes the meaning of a given category can only be guessed from its terms and its place in the hierarchy. Wherever the meaning guessed from the concept name of a category differs from the meaning deduced from the hierarchy translation errors may arise. Such risk could be decreased by natural language descriptions of all categories, which would also facilitate the ontological analysis.

Apart from the consistency problems coming from the integration of other conceptual systems, another question arises: is it reasonable to import an even consistent category from some existing medical classification just because it is there? If every major classification system were imported into SNOMED CT, the resulting terminology would be too large to be manageable. It would be overcrowded by clinically irrelevant terms that often refer to artificial concepts (like the well-known "*classified elsewhere*" concepts). The worst case is given when not only the categories but also the consistency errors are imported without care.

Even those things that exist in the real world (and as such are potentially relevant for medicine) should be carefully filtered according to their practical importance. In that sense, SNOMED CT seems to be heterogeneous. It contains concepts such as *Mars bar *and *Kit Kat*, whereas it would suffice to list chocolate candy, without its children. IHTSDO inactivated these unnecessary concepts in later versions. Nevertheless, SNOMED CT still contains *unidentified flying object*, classified as a transport vehicle that is at least debatable. On the other hand, there is a concept *tendon pulley reconstruction *but the related anatomical concept tendon pulley is missing.

## Conclusion

As a conclusion, there are at least three possible options regarding the potential use of SNOMED CT:

i. Use of SNOMED CT as a plain or loosely structured list of terms that perhaps requires some extension of the coverage but does not need substantial restructuring. The resulting system could serve as a common terminology supporting a limited interoperability, but could not be efficiently used for intelligent services.

ii. To restructure SNOMED CT into a high-quality ontology. It should be modularised, with clear separation of

• A formal top level ontology (e.g. DOLCE).

• A high level core reference ontology of shared medical knowledge (anatomy, physiology, pathology and medical procedures, etc.).

• A set of (sub)domain ontologies of the different medical specialities.

As a first step, the core model should be aligned to the top-level ontology. Roles have to be separated from the concepts to allow logic-based representation. During the building of the core model, each category should be carefully analysed to be inserted into the correct place in the hierarchy. Manually asserted multiple inheritances should only be kept in exceptional cases. The formalisation makes possible the automated classification of multiple inheritances and also enables consistency checking.

Whenever possible compound entities in the domain specific extensions should be formally defined based on the concepts of the core ontology. However, we expect a significant number of medical concepts that cannot be represented in such a way (typically due to the lack of necessary knowledge about the given entity, such as autism). These concepts remain manually asserted into the hierarchy under the nearest formally defined concept.

iii. The third option is to build a new medical ontology from scratch (including the partial reuse of existing ones), and to restrict the use of SNOMED CT for interoperability by mapping concepts to it. The authors already performed such experiments targeting a core anatomy model derived from the Foundational Model of Anatomy [12]. In a similar way, parts of SNOMED CT can also be reused.

As we focus our research on the formal representation of classification systems, a conceptual system appropriate for formal reasoning is required. Therefore, option (i) can be ruled out. The final decision between option (ii) and (iii) will require a more thorough analysis. Option (ii) requires international co-operation, possibly within the IHTSDO, if the adoption of formal rigour gains wide support.

## Competing interests

The authors declare that they have no competing interests.

## Authors' contributions

GH conceived of the analysis, carried out the concept selection and analysis, and drafted the manuscript. GS conceived of the analysis and helped to draft the manuscript. PV provided the DOLCE-based classification of concepts. All authors read and approved the final manuscript.
